# Efferocytosis: a therapeutic strategy for diabetes and its vascular complications

**DOI:** 10.3389/fcell.2025.1641748

**Published:** 2025-11-10

**Authors:** Yi Song, Jiayi Yuan, Yifan Li, Yifan Liu, Guijun Qin

**Affiliations:** 1 Department of Endocrinology and Metabolism, The First Affiliated Hospital of Zhengzhou University, Zhengzhou, China; 2 The First Clinical Medical College of Zhengzhou University, Zhengzhou, China; 3 Department of Pediatric Transplantation, Tianjin First Central Hospital, Tianjin, China

**Keywords:** efferocytosis, diabetes, diabetic vascular complications, inflammation, immunology, efferocytosis-based therapy

## Abstract

**Purpose:**

Efferocytosis is a continuous multistep process that efficiently clears dead cells and is essential for maintaining homeostasis in multicellular organisms. Under normal circumstances, apoptotic cells release “find-me” and “eat-me” signals that stimulate their engulfment and clearance. However, in many chronic inflammatory diseases, the clearance of apoptotic cells is significantly impaired. This review explores the relationship between defective efferocytosis and diabetes-related vascular complications, while investigating underlying pathophysiological mechanisms to identify novel therapeutic targets for disease management.

**Methods:**

After searching in PubMed and Web of Science databases using ‘efferocytosis’, ‘diabetic vascular complications’, and ‘efferocytosis-based therapy’ as keywords, studies related were compiled and examined.

**Results:**

This review summarizes the specific process of phagocytes engulfing and clearing dead cells, and explains the development of diabetes and its vascular complications from the perspective of defects in efferocytosis.

**Conclusion:**

The review points out that molecules and pathways associated with efferocytosis are potential targets for treating diabetic vascular complications, providing new ideas for clinical treatment.

## Highlight

•This review illustrates the process of efferocytosis.
•Defects in efferocytosis can lead to homeostatic imbalance, resulting in the onset of diseases.
•The review points out that molecules and pathways associated with efferocytosis are potential targets for treating diabetic vascular complications, providing new ideas for clinical treatment.

## 1 Introduction

Statistically, billions of cells die every day and are replaced by newly generated cells, which is important for development (Bertheloot et al., 2021). Dead cells are efficiently cleared by phagocytes through efferocytosis, a crucial process for maintaining balance in multicellular organisms. Efferocytosis is executed by three different categories of phagocytic cells: professional, non-professional, and specialized. Professional phagocytes, such as macrophages, dendritic cells, Kupffer cells in the liver, and microglial cells in the nervous system, are the main phagocytes responsible for efferocytosis. They can rapidly and efficiently engulf and clear apoptotic cells (ACs), enabling multicellular organisms to recycle cellular components (Lazarov et al., 2023; Mass et al., 2023). Non-professional phagocytes also demonstrate high phagocytic efficiency, which can engulf and eliminate ACs under specific conditions. For example, airway epithelial cells, intestinal epithelium, and mammary epithelial cells can clear neighboring apoptotic epithelial cells (Poon et al., 2014; Akhtar et al., 2016; Blander, 2016). Specialized phagocytes, classified as tissue-resident phagocytes, serve crucial homeostatic and cytoprotective functions within tissues while also orchestrating targeted phagocytic clearance under specific physiological conditions, as exemplified by retinal pigment epithelial cells and Sertoli cells in the testes (Penberthy et al., 2018; Kwon et al., 2020).

Efferocytosis play a vital role in maintaining tissue health and resolving inflammation. During development, organ structure, limb formation, and the negative selection of T cells necessitate that cells undergo physiological apoptosis. However, since ACs can undergo secondary necrosis and trigger immune responses, it is essential to remove them rapidly in a non-inflammatory manner. Phagocytosis is a process in which the engulfed cell uptake of extracellular particles larger than 0.5 μm in diameter (Moon et al., 2023).​ Phagocytosis mitigates inflammation by stimulating the production of anti-inflammatory cytokines while inhibiting pro-inflammatory cytokines, thereby promoting the resolution of inflammation. These processes occur through integrated yet mechanistically distinct pathways (Doran et al., 2020). Efferocytosis offers three main benefits: Firstly, the efficient clearance of ACs can prevent secondary necrosis of these cells, which would otherwise lead to secondary inflammation and chronic diseases (Lauber et al., 2004). Secondly, the removal of ACs provides space for the growth and development of metabolically active stem cells and ample nutrients (Okabe and Medzhitov, 2016). Thirdly, during efferocytosis, phagocytic cells induce an anti-inflammatory process by increasing the secretion of anti-inflammatory mediators and upregulating transcriptional programs that promote repair, leading to the production of immunosuppressive molecules (Arandjelovic and Ravichandran, 2015). Obstacles to the clearance of cellular debris and defects in efferocytosis can lead to various diseases, including atherosclerosis, diabetes and obesity, aging, cancer, infectious diseases, systemic lupus erythematosus, neurodegenerative diseases, retinal degeneration, and rheumatoid arthritis (Boada-Romero et al., 2020; Mehrotra and Ravichandran, 2022).

Diabetes is a health concern growing at an alarming rate worldwide, emerging as another global health crisis in the 21st century (InternationalDiabetesFederation, 2021). In fact, chronic low-grade inflammation constitutes a characteristic of type 2 diabetes mellitus (T2DM), and patients with T2DM have higher levels of pro-inflammatory cytokines (Lontchi-Yimagou et al., 2013), such as tumor necrosis factor-alpha (TNF-α), interleukin-1β (IL-1β), and interleukin-6 (IL-6), which are not only associated with insulin resistance and beta cell dysfunction, but also intricately linked to the occurrence and development of cell clearance (van Poppel et al., 2014; Boada-Romero et al., 2020). Diabetic vascular complications have a high mortality rate. Their development is closely associated with inflammation, primarily manifesting as endothelial dysfunction and atherosclerosis (Li Y. et al., 2023). Interestingly, efferocytosis is an anti-inflammatory process that produces immunosuppressive molecules (Arandjelovic and Ravichandran, 2015), and research has already shown the close association between efferocytosis and diabetic vascular complications (Kanter et al., 2020; Rawal et al., 2024; Gardiner et al., 2025; Li et al., 2025). Therefore, we systematically review the general mechanisms of efferocytosis and the role of efferocytosis failure in T2DM-associated vascular complications, to establish a conceptual framework for developing mechanistically targeted therapeutic interventions.

## 2 The mechanisms of efferocytosis

**FIGURE 1:**
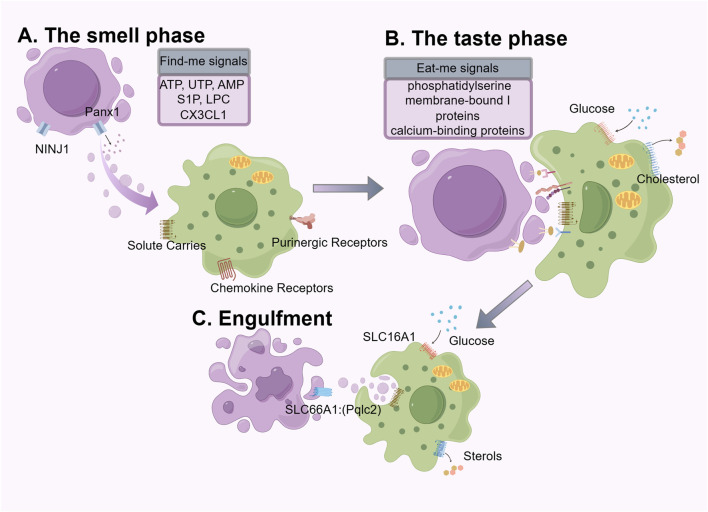
The mechanics of efferocytosis is a continuous multistep process that involves the “smell phase”, “taste phase”, and “engulfment and digestion phase”, each of which involves different molecules and signals. We use purple to label apoptotic cells and green to label phagocytes.

The removal of ACs through efferocytosis is a continuous multi-step process. The first step is the “smell phase”, during which phagocytes “sense” the presence of ACs and locate them. The second step is the “taste phase”, during which the ligands on ACs bind to phagocytic receptors on phagocytes, allowing them to better localize to the dying cells for the subsequent engulfment step. The third step is the “engulfment and digestion phase”, during which the phagocytes ingest and process the ingested corpses and their contents. (Figure 1).

### 2.1 The smell phase

The smell phase is the first step to successful efferocytosis. Common forms of cell death include apoptosis, necroptosis, pyroptosis, and ferroptosis. Under steady-state conditions, cell apoptosis induced by gene-programmed suicide mechanisms is the most common (Newton et al., 2024). Unlike the pro-inflammatory response triggered by pathogen engulfment, the engulfment of ACs typically induces an anti-inflammatory response or immune tolerance (Zhang et al., 2019; Medina et al., 2020; Ampomah et al., 2022; Moon et al., 2023). Cells undergoing apoptosis and non-apoptotic cell death display and release soluble mediators, emitting various “find-me” signals to guide subsequent phagocytic processes. The forms of “find-me” signals vary, including nucleotides (ATP, UTP, AMP) (Elliott et al., 2009; Chekeni et al., 2010), membrane lipids (lysophosphatidylcholine (LPC), lysolecithin) (Lauber et al., 2003), chemokines (CX3CL1) (Truman et al., 2008). The release of nucleotides is closely related to the progression of apoptosis; the pan-connexin channel on the apoptotic cell membrane is cleaved and activated by caspase 3/7, leading to the release of ATP and UTP (Elliott et al., 2009). During the early stages of apoptosis, apoptotic cells (AC) release small amounts of nucleotides, which can serve as potent inducers for phagocyte migration towards the AC. Upon complete lysis of apoptotic cells, high concentrations of intracellular ATP are released (Mehrotra and Ravichandran, 2022). It is noteworthy that high levels of extracellular ATP can trigger a robust inflammatory response, during which other pathways are activated to promote a tolerogenic response when exposed to low doses of nucleotides for a long term (la Sala et al., 2003; Cauwels et al., 2014; Schilperoort et al., 2023). One such pathway involves the upregulation of the chloride transporter SLC12A2, which has been reported to inhibit the pro-inflammatory signaling induced by apoptotic cells (Perry et al., 2019). CX3CL1 protein belongs to indirect signaling molecules, rapidly released from ACs through caspase-3 and bcl-2-dependent regulation, and subsequently interacts with fractalkine receptors on macrophages to be attracted to apoptotic sites (Truman et al., 2008).

LPC and Sphingosine 1-phosphate (S1P) are apoptotic-specific discovery signals. Following apoptosis, activated caspase-3 mediates the activation of calcium-independent phospholipase A2, causing ACs to release “chemical attractants” such as lysolecithin (Lauber et al., 2003). S1P is produced by sphingosine via sphingosine kinase and regulates the chemotactic activity of phagocytic cells by participating in the G protein-coupled receptor signaling (Garris et al., 2013).

The actions of these soluble mediators are closely related to the types of phagocytes and ACs (Elliott et al., 2017). On one hand, they serve as attraction signals, recruiting phagocytes to migrate towards ACs. On the other hand, they stimulate the clearance potential of phagocytes, enhance the expression of cell surface phagocytic receptors, regulate cell cytoskeletons, and prepare for subsequent phagocytosis adequately (Medina et al., 2020). High efficiency in the release of find-me signals, sensitive signal perception, and the non-abnormal degradation of find-me signals are all crucial for the recognition and clearance of ACs.

### 2.2 The taste phase

The taste phase is the second step of efferocytosis. The accurate recognition and processing of ACs by phagocytes rely on two key factors: “eat me” signals on the surface of ACs and “do not eat me” signals on the surface of healthy viable cells (Kelley and Ravichandran, 2021), as well as specific phagocytic receptors on phagocytes (Arandjelovic and Ravichandran, 2015).

The most important “eat me” signal is phosphatidylserine (PS), which is evolutionarily conserved. Under normal physiological conditions, PS is usually restricted to the inner leaflet of the cell membrane by “flippases”. However, during stress or apoptosis, caspase three in ACs causes the breakdown and inactivation of flippases, leading to the activation of “scramblases”, which rapidly expose PS on the cell surface. PS is then recognized by ligands on adjacent phagocytes for engulfment (Suzuki et al., 2013; Segawa and Nagata, 2015). Phagocytes possess two types of ligand-receptor interactions for recognizing ligands and PS receptors: direct receptors and indirect receptors, which vary according to the ligand molecule involved. BAL1(Sokolowski et al., 2011), TIM(Kobayashi et al., 2007; Miyanishi et al., 2007; DeKruyff et al., 2010), and Stabilin2 (Lee et al., 2011) can directly bind to PS, while the receptor tyrosine kinase family TAM Receptors (Tyro3, Axl, and Mertk) (Lemke, 2017; Burstyn-Cohen and Fresia, 2023) bind to PS indirectly through soluble bridging molecules such as Gas6 and Protein S. PS, a lipid membrane component, enhances ligand binding affinity and facilitates TAM receptor-mediated signaling (Nakano et al., 1997; Graham et al., 2014; Zagórska et al., 2014; Kasikara et al., 2017). This effect is achieved through the calcium-dependent binding of PS to the Gla domain of either Gas6 or Protein S (Nelsestuen et al., 1978; Nakano et al., 1997). In this mechanism, Gas6 and Protein S function as bridging ligands that connect PS with the TAM receptor. The inclusion of PS-containing lipid membranes alongside TAM receptor ligands results in elevated receptor phosphorylation relative to the application of ligand alone (Zagórska et al., 2014; Kasikara et al., 2017). Such bridging-mediated signaling plays a critical role in the phagocytic clearance of apoptotic cells that externalize PS. In addition to PS, common “eat-me” signals on ACs include membrane-bound I proteins (Arur et al., 2003) and calcium-binding proteins (Gardai et al., 2005). Studies have shown that aside from serving as “find me” signal molecules, LPC on the membrane of dying cells can also interact with receptors on phagocytes (Kim et al., 2002). It is worth mentioning that MerTK and AXL play a vital role in anti-inflammation response and establishing tolerance to “self” antigens (Lemke and Rothlin, 2008). During the period of efferocytosis, MerTK functions to reduce inflammatory cytokine production and M1-like macrophage polarization through the inhibition of the NF-κB pathway (Tibrewal et al., 2008). Beyond suppressing the M1-like phenotype, MerTK also ​promotes​ macrophage ​differentiation​ into the alternative, reparative M2-like phenotype (Filardy et al., 2010; Zizzo et al., 2012). The expression and activation of MerTK promotes T cell tolerance by inhibiting the maturation of dendritic cells and the expansion of effector T cells (Wallet et al., 2008).

Healthy neighboring cells express “do not eat me” signals on their surface, which help avoid the unnecessary clearance of viable “healthy” cells (Kelley and Ravichandran, 2021). The cell surface proteins CD47 and CD24 are two classic signals that are recognized by the receptors SIRPα on macrophages and SIGLEC-10, respectively (Oldenborg et al., 2000; Barkal et al., 2019). CD47 is a crucial “do not eat me” molecule. However, upregulation of CD47 expression on diseased cells can lead to defects in phagocytosis and the occurrence of inflammation (Kojima et al., 2016).

### 2.3 Engulfment

The third step of efferocytosis involves the ingestion and degradation of dying cells by engulfing cells, which specifically includes the following steps: Ingestion of dying cells, degradation by lysosomes, maturation of phagosomes, and phagosome dissolution (Boada-Romero et al., 2020). The ingestion of dying cells occurs after engulfing cells recognize the dying cells, leading to the internalization of the dying cells through downstream signaling of AC engulfment mediated by engulfment receptors, forming “phagosomes (Levin et al., 2016). Actin polymerization is a critical component of phagosome formation and efficient capture of dead cells, while actin depolymerization is equally important for the detachment of phagosomes from the plasma membrane. The cargo ingested by phagocytes poses a metabolic burden, including amino acids, lipids, and nucleic acids. These metabolic byproducts expose phagocytes to a plethora of potentially cytotoxic macromolecules, necessitating their safe and efficient handling (Han and Ravichandran, 2011). As a mechanism for handling excess lipids within macrophages, the cholesterol transporter ATP-binding cassette transporter A1 (ABCA1) is upregulated following the detection of apoptotic cells during the feeding phase, facilitating the efflux of cholesterol to apolipoprotein A1 (Fond et al., 2015; Viaud et al., 2018). Phagosomes fuse with lysosomes to form “phagolysosomes”, which contain various proteases, lipases, and nucleases to digest the cargo of phagosomes to maintain homeostasis (Levin et al., 2016; Boada-Romero et al., 2020). In terms of energy metabolism, glycolysis within phagocytes facilitates actin polymerization and sustained uptake of dying cells. Sustained exposure to apoptotic cellular debris, phagocytes can efficiently undergo serial efferocytic processing to optimize efferocytosis, particularly crucial in tissues with high cell turnover (Trzeciak et al., 2021). Metabolites derived from AC metabolism in macrophages can achieve optimal sustained efferocytosis and resolution of injury (Yurdagul et al., 2020). Typical phagocytic pathways include the maturation from early to late phagosomes, ultimately fusing with lysosomal compartments. There is also an additional pathway involving microtubule-associated protein 1A/1B light chain 3 (LC3)-associated phagocytosis (Green et al., 2016). LC3-associated phagocytosis promotes the assembly and acidification of phagolysosomes (Martinez et al., 2011).

Upon completion of mature modifications, phagolysosomes begin to degrade AC remnants and release anti-inflammatory cytokines such as IL-10 (Xu et al., 2006), TGF-β (Fadok et al., 1998; Freire-de-Lima et al., 2006), and specialized pro-resolving lipid mediators (SPMs), including lipoxin A4, resolvin D1, D2, and E2 (Basil and Levy, 2016); simultaneously reducing the expression of pro-inflammatory prostaglandins and leukotriene B4, Lipoxin A4 and resolvin D1 further enhance phagocytosis, establishing a feedback loop that increases their own production (Dalli and Serhan, 2016; Kourtzelis et al., 2020). Phagocytosis promotes a pro-resolving phenotype by downregulating the expression of pro-inflammatory cytokines (Godson et al., 2000), increasing the levels of pro-resolving mediators, inhibiting inducible nitric oxide synthase (iNOS), and enhancing the production of vascular endothelial growth factor (VEGF) (Doran et al., 2020). Macrophages polarized towards a pro-resolving ARG1+ phenotype, which are more efficient phagocytes compared with pro-inflammatory macrophages (Yurdagul et al., 2020).

Recent studies utilizing murine models of T2DM have demonstrated defective efferocytosis in peritoneal macrophages, bone marrow-derived macrophages, and wound macrophages (Li et al., 2009; Khanna et al., 2010; Suresh Babu et al., 2016). In individuals with diabetes, macrophages undergo functional alterations characterized by heightened responsiveness to inflammatory stimuli and enhanced secretion of pro-inflammatory mediators. Concurrently, their phagocytic capacity for pathogens and efferocytic clearance of apoptotic cells are impaired. These deficits compromise pathogen elimination and, due to the role of efferocytosis as an inducer of phenotypic switching, result in a decreased population of M2-like reparative macrophages (Mao et al., 2025).

## 3 Defective efferocytosis represents a novel mechanism that promotes the development of vascular complications in diabetes

**FIGURE 2:**
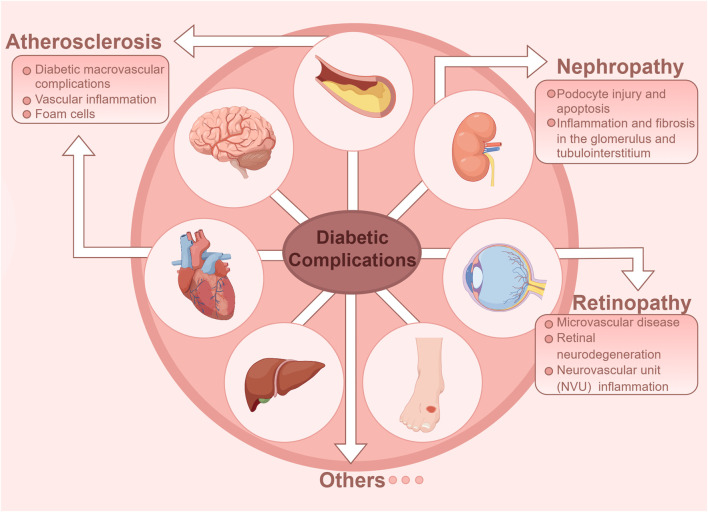
Diabetic vascular complications in diabetes are classified as either macrovascular or microvascular disease. Macrovascular complications include atherosclerosis of large and medium-sized arteries, while microvascular complications primarily affect the retina and kidneys.

As a common metabolic disorder, persistently elevated blood glucose level in diabetic patients can lead to dysregulated inflammation. High glucose levels trigger monocyte/macrophage infiltration into pancreatic islets and insulin-sensitive tissues, ​thereby polarizing​ macrophages toward a pro-inflammatory phenotype (Ying et al., 2020). Abundant researches have confirmed that cytokines and pro-inflammatory lipids play a crucial role as drivers of inflammation. However, current therapeutic interventions for diabetic vascular complications remain suboptimal, with a paucity of effective treatment modalities available (Tomic et al., 2022). The clearance of endogenous lipid mediators, such as SPMs, can promote the resolution of inflammation and alleviate microvascular and macrovascular complications of diabetes (Basil and Levy, 2016; Kwon et al., 2016; Zatterale et al., 2019). In addition, cytokines and pro-inflammatory lipid mediators play important roles in the physiological mechanism of efferocytosis. We have synthesized the pathophysiological mechanisms linking defective efferocytosis to the pathogenesis of diabetes and associated vascular complications, while proposing novel therapeutic avenues targeting efferocytic pathways for clinical intervention (Figure 2).

### 3.1 Macrovascular complication of diabetes: atherosclerosis

Diabetic macrovascular complications include atherosclerosis of large arteries and medium arteries, which is a common pathological feature of vascular disease in diabetic patients (Li Y. et al., 2023). Atherosclerotic cardiovascular disease (CVD) is the leading cause of death in adult diabetic patients (Kanter et al., 2020). High blood sugar is a significant risk factor for atherosclerosis, promoting endothelial dysfunction, and is considered an early event in the development of atherosclerotic lesions (Demir et al., 2021). Atherosclerosis is understood as a cholesterol storage disease and a lipid-driven inflammatory condition (Bäck et al., 2019). Hyperglycemia not only induces oxidative stress and modifies LDL-C into oxLDL form (Evans et al., 2002), but also promotes macrophage epigenetic and metabolic reprogramming, accelerating the inflammatory response (Russo et al., 2021). A study in mice demonstrated that the absence of low-density lipoprotein receptors leads to impaired phagocytosis of lesions and a larger necrotic core compared to healthy animals (Li et al., 2009). In the initial stage of atherosclerotic lesion development, circulating monocytes adhere to endothelial cells and enter the vessel wall, then differentiate into macrophages. These macrophages absorb excess oxLDL via scavenger receptors and become lipid-laden foam cells (Rawal et al., 2024), which must be cleared via efferocytosis. Inefficient clearance of dead cells is a major factor in the progression of atherosclerosis (Boada-Romero et al., 2020).

Defective efferocytosis is commonly considered the cause of improper clearance of ACs. Defective efferocytosis contributes to plaque expansion, plaque rupture, acute coronary syndrome, and stroke (Vucic et al., 2012; Poon et al., 2014). The NLRP3 inflammasome regulates vascular inflammation and atherosclerosis by modulating efferocytosis (Rochette et al., 2023). The NLRP3 inflammasome is a multimolecular complex that functions at the molecular level to mediate caspase-1 activation. Upon stimulation by various pathogen-associated molecular patterns (PAMPs) or DAMPs, it induces the release of interleukin-1β (IL-1β) and interleukin-18 (IL-18) within cells (Duewell et al., 2010). During the early pro-inflammatory stage of atherosclerosis, elevated TNF-α hinders the timely clearance of ACs, weakening efferocytic activity (Martinet et al., 2011). Additionally, mice lacking TIM-4, Mertk, MFGE8, or Pros exhibit failed AC clearance (Ait-Oufella et al., 2007; Cai et al., 2017), and increased inflammation has been observed (Kimani et al., 2014). EC is a unique barrier to inflammation (Shao et al., 2020). High levels of PCSK9 expression are observed in vascular cells, comprising endothelial cells, smooth muscle cells, and macrophages (Ding et al., 2015a; Ding et al., 2015b; Ding et al., 2020). Research has found that proprotein convertase subtilisin/kexin type 9 (PCSK9) levels increase in aortic endothelial cells during aging, which is associated with the loss of the phagocytic receptor MerTK. MerTK serves as a critical receptor for efferocytosis and is highly expressed in endothelial cells (ECs), indicating their potential capability to mediate efferocytosis (Li et al., 2019). Furthermore, the excessive production of reactive oxygen species (ROS) and chronic inflammation, characteristic of vascular aging, is alleviated in the aortas of PCSK9-deficient mice (Liu et al., 2023). Additionally, considering that PCSK9 promotes the clearance of oxidized LDL, subsequently increasing the synthesis of LDL receptors, the use of PCSK9 inhibitors can lower LDL concentrations in patients with diabetes (Chen et al., 2023). The factors exacerbate the progression of diabetic macrovascular complications by affecting different factors in the efferocytosis process.

### 3.2 Microvascular complications of diabetes

Diabetic microvascular disease is caused by microvascular lesions in arterioles, capillaries, and venules. Microvascular disease can cause pathological and functional changes in many tissues, which are traditionally referred to as diabetic retinopathy (DR), diabetic kidney disease (DKD), diabetic cardiomyopathy (DCM), peripheral neuropathy, and autonomic neuropathy (Lu et al., 2023; Wang et al., 2023). It is noteworthy that DKD is the leading cause of end-stage renal disease, while DR is one of the major causes of blindness among various eye diseases. Unfortunately, due to the complex pathogenesis of these two diseases, effective treatment options are still lacking. Multiple studies have indicated that hyperglycemic states, inflammatory reactions, and macrophages play crucial roles in the development and progression of DKD, DR and DCM. Therefore, we have focused our review on the related mechanisms of inflammation and efferocytosis in diabetic microvascular disease, and look forward to future research and treatment prospects (Li Y. et al., 2023).

#### 3.2.1 Diabetic kidney disease

Studies have shown that DKD is a chronic low-grade inflammation in the kidneys. The abnormal infiltration of inflammatory cells in DKD kidney, including macrophages, dendritic cells, T lymphocytes, B lymphocytes, and neutrophils, is an important pathological process that mediates kidney injury. Their pathologically amplified cellular crosstalk with intrinsic kidney cells, such as renal tubular epithelial cells, glomerular endothelial cells, and podocytes, exacerbates kidney injury (Rayego-Mateos et al., 2023). In addition, multiple studies have reported that the accumulation of intrinsic kidney cells that undergo various forms of cell death (apoptosis, pyroptosis, ferroptosis, necrosis, and autophagic cell death) is an important factor in the chronic inflammation of DKD (Jianbing et al., 2022; Jiang et al., 2022; Wang et al., 2022; Song et al., 2023a).

Our previous studies have indicated that RAC1 is a key regulator of efferocytosis, which can promote macrophage phagocytosis of renal tubular cells and reduce inflammation response in DKD (Song et al., 2023b). Other studies have indicated that tctex-1 promotes renal tubular epithelial cell efferocytosis and improves acute kidney injury by binding to kidney injury molecule-1 (Ismail et al., 2018). On the other hand, JAML inhibits macrophage efferocytosis through C-type lectin and promotes the inflammatory response in acute kidney injury (Huang et al., 2022).

In the kidney, glomerular endothelial cells (GEnCs), as specialized vascular cells, form the walls of glomerular tufts and play an important role in maintaining kidney homeostasis. Endothelial dysfunction can increase endothelial permeability and apoptosis, leading to the loss of GEnCs' fenestration function and resulting in proteinuria (Mohandes et al., 2023). Studies have shown that the severity of glomerular endothelial cell injury is closely related to the heterogeneity of macrophage infiltration in capillary proliferative glomerulonephritis (EP) (Arai et al., 2021). Although the interaction between macrophages and glomerular endothelial cells is ubiquitous in renal tissues, it is still unclear whether macrophages participate in phagocytosing and clearing glomerular endothelial cells.

On the other hand, the activation of macrophages can promote podocyte injury and apoptosis (Guo et al., 2017; Ji et al., 2019; Li et al., 2022), as well as inflammation and fibrosis in the glomerulus and tubulointerstitium (Tang et al., 2019; Song et al., 2023b). T cell immunoglobulin domain and mucin domain-3 (Tim-3) is a PS receptor in “the taste phase”, which elevates the activation of nuclear factor κB (NF-κB)/TNF-α signal in macrophage, promoting podocyte injury in diabetic mice (Yang et al., 2019), but whether it participates in efferocytosis remains to be studied.

Autophagy is a conserved lysosomal pathway for the degradation of cytoplasmic components (Dikic and Elazar, 2018). Basal autophagy in renal cells is crucial for maintaining renal homeostasis, structure, and function. Dysregulated autophagy contributes to the pathogenesis of DKD. Emerging evidence highlights that microtubule-associated protein LC3-mediated autophagy serves as a critical efferocytic mechanism, wherein the sustained membrane localization of LC3 is indispensable for autophagosome elongation and membrane closure in diabetic kidney disease (Wei et al., 2018; Lystad and Simonsen, 2019). Studies have shown that in diabetic mice, p62 is upregulated, while LC3-II is downregulated, indicating that autophagic activity is inhibited, which affect efferocytosis process, promoting inflammatory response in kidney (Gui et al., 2019; Liu et al., 2021). Study has shown that in LC3-mediated autophagy, SPMs are critical mediators released by phagocytic cells during the degradation of apoptotic cells, which can accelerate the resolution of inflammation (Motwani et al., 2016; Goli et al., 2025). Significantly, SPMs also exert protective effects on resident cell populations in local tissues, including mesangial cells, podocytes, and renal tubular epithelial cells, as well as vascular smooth muscle cells and endothelial cells. SPMs with pro-resolving bioactions include attenuating endothelial cell activation, blocking neutrophil extravasation, promoting non-phlogistic monocyte recruitment, inducing an M2 resolving macrophage phenotype, and stimulating neutrophil apoptosis and macrophage efferocytosis (Börgeson and Godson, 2010; Brennan et al., 2021). These provide new research targets for the treatment of DKD.

#### 3.2.2 Diabetic retinopathy

DR is a common complication of diabetes that can lead to visual impairment and blindness (Flaxman et al., 2017). Substantial evidence derived from both clinical studies and experimental animal models demonstrates that DR represents a chronic low-grade inflammatory condition mediated by sustained hyperglycemia. (Noda et al., 2012), and sustains by cytokines such as IL-6, IL-8, and monocyte chemoattractant protein-1 (MCP-1, also known as C-C motif chemokine ligand 2 (CCL2)) (Ambrosini and Aloisi, 2004; Forrester et al., 2020; Yue et al., 2022). Interestingly, these inflammatory cytokines and adhesion molecules belong to damage-associated molecular patterns (DAMPs) (Hilgendorf et al., 2024), which can act as both inducers of inflammation and chemical attractants for macrophages (Boada-Romero et al., 2020).

Retinal pigment epithelial (RPE) cells are the primary phagocytic cells in the eye, and their diurnal phagocytosis and clearance is a conserved cellular ingestion process. The research indicates that dysfunction of the RPE can lead to proliferative diabetic retinopathy (PDR) (Strauss, 2005; Joussen et al., 2007; Stitt et al., 2016). The retinal pigment epithelium (RPE) plays a crucial role in the phagocytosis and subsequent digestion of photoreceptor outer segments (POS), which is essential for maintaining the function and survival of photoreceptors. The phagocytic process of POS is regulated by the MERTK protein (Feng et al., 2002).

MERTK, as a type of receptor tyrosine kinase (RTK), belongs to the TAM family, which also includes Tyr3 and Axl. The key role of MERTK in physiological processes is to mediate the phagocytosis of dying and dead cells. MERTK can be cleaved by ADAM9, releasing a soluble form known as sMer. Macrophages exposed to high glucose levels exhibit elevated sMer levels, which leads to a reduction in phagocytosis. The presence of sMer hinders the binding between POS and RPE, while inhibiting MERTK cleavage enhances this binding, resulting in increased phagocytosis (Feng et al., 2002). It is worth noting that MERTK has been identified as a key disease-causing gene in inherited retinal dystrophy, but whether it functions through the macrophage efferocytosis remains unclear (D'Cruz et al., 2000; Boada-Romero et al., 2020).

Additionally, it is worth mentioning that the most typical feature of PDR is the imbalance between ischemia-mediated angiogenic factors and anti-angiogenic factors, leading to neointimal proliferation and subsequent vitreous hemorrhage and retinal detachment. This may be related to the accumulation of neointima after ineffective clearance by phagocytic cells, although confirmatory experimental validation is required to substantiate this mechanistic hypothesis (Li H. et al., 2023). Research has shown that in the context of atherosclerosis ineffective efferocytosis leads to the accumulation of necrotic and pyroptotic cells, releasing DAMPs such as HMGB1 and ATP, and inducing vascular permeability (Gerlach et al., 2020). This may induce macrophages to migrate to eye tissues, phagocytose type II collagen that is normally isolated from the immune system, and activate B cells to produce anti-type II collagen antibodies (Catchpole et al., 2001), leading to the development of diabetic macular edema. These findings suggest that the pathogenesis of diabetic retinopathy is closely associated with a defect in the clearance of dead cells.

#### 3.2.3 Diabetic cardiomyopathy and delayed wound healing

Diabetic cardiomyopathy and delayed would healing, as both complications are known to be associated with chronic inflammation and defective cell clearance (Karnam et al., 2020; Zhang et al., 2024). Diabetic cardiomyopathy is a cardiac microvascular disorder caused by diabetes mellitus. It is distinct from hypertensive heart disease, coronary atherosclerotic heart disease, valvular heart disease, and other cardiac pathologies (Wang et al., 2023). The primary risk factor for the progression of diabetic cardiomyopathy is the progressive death of cardiomyocytes (Filardi et al., 2019) and alterations in endothelial cells (Knapp et al., 2019) under hyperglycemic conditions. In individuals with diabetes, the hyperglycemic environment triggers cardiomyocyte injury. Necrotic cardiomyocytes then release DAMPs, which activate mast cells and stimulate the secretion of TNF-α and IL-1β. This cascade ultimately leads to endothelial cell activation (Zhang et al., 2024). The impaired wound healing in diabetic patients involves multifactorial pathophysiology, including persistent inflammation, compromised angiogenesis, and dysfunctional cellular responses (Armstrong et al., 2023). Vascular complications in diabetic patients can impair wound healing and contribute to the development of diabetic foot ulcers (Brem and Tomic-Canic, 2007). Diabetic patients often exhibit impaired macrophage function, characterized by diminished capacity to clear infections and pathogens, as well as defective efferocytosis, which collectively contribute to delayed wound healing and increased risk of amputation (Aitcheson et al., 2021).

## 4 Efferocytosis-based therapeutic opportunities in diabetes

Therapeutic augmentation of efferocytosis demonstrates significant potential in attenuating pathological progression across multiple disease states. Therefore, efferocytosis-related molecules and pathways are potential targets for treating diabetic vascular complications, providing new insights for clinical therapy. The development of novel treatment approaches targeting efferocytosis is highly worthwhile for discussion. (Table 1).

**TABLE 1:** Efferocytosis-based therapeutic opportunities.

Category	Therapy	Targeted molecules	References
Commonly used medications	statins	CD47	N. Eberhardt (2022), Tajbakhsh et al. (2022)
	statins	Ras homolog gene family member A	Salman et al. (2008)
	MR antagonists (spironolactone)	mineralocorticoid receptor (MR)	Xie et al. (2022)
	p38 inhibitor (losmapimod)	TIM-4	Anand et al. (2011), Patnaik et al. (2016), De Maeyer et al. (2020)
Biologic agents	CD47 antibodies	CD47	Chen et al. (2021)
	PD1-/PD-L1 antibodies	PD1-/PD-L1	Chen et al. (2021)
Nanotechnology	siRNA against CaMKIIγ	MerTK	Flores et al. (2020)
	CD47-SIRPα signaling inhibitors	CD47	Flores et al. (2020)

### 4.1 Commonly used medications that enhance efferocytosis

Several commonly used medications can enhance the efferocytosis of macrophages. Defective efferocytosis is considered a major risk factor for atherosclerosis, and enhancing efferocytosis can help reduce atherosclerotic lesions. Recent investigations have revealed that CD47, a critical ‘don’t-eat-me' signaling molecule, is paradoxically overexpressed across a spectrum of cancers (Majeti et al., 2009; Chao et al., 2011; Willingham et al., 2012). This upregulation enables malignant cells to evade established immune surveillance mechanisms, including elimination by tumoricidal macrophages, and is now considered a key mechanism underlying tumor progression (Perisic et al., 2013; Kojima et al., 2019). Studies suggest that statins increase macrophage “eat me” signals and induce a reduction in the “do not eat me” signal CD47 (N. Eberhardt, 2022; Tajbakhsh et al., 2022); On the other hand, statins can lower cholesterol and inflammation, inhibiting the overexpressed Ras homolog gene family member A, which is a negative regulator of phagocytosis in atherosclerotic lesions (Salman et al., 2008). The mineralocorticoid receptor (MR) is considered a potential target for the treatment of atherosclerosis. MR deficiency in apoE^−/−^ mice reduced the number of ACs in atherosclerotic lesions and increased effective efferocytosis, suggesting that MR antagonists like spironolactone could improve macrophage efferocytosis (Xie et al., 2022). The impairment of the p38 MAPK signaling axis is considered a fundamental mechanism underlying the reduced expression of TIM-4 in mononuclear phagocytes of the elderly, leading to compromised cytoplasmic function and inflammation resolution. To address this damage, the p38 inhibitor losmapimod is currently undergoing clinical trials. Losmapimod blocks p38 and restores TIM-4 expression and efferocytic activity in elderly mononuclear phagocytes under *in vitro* conditions. Elderly patients receiving oral losmapimod show improved resolution of skin inflammation, with a reduction in unresolved polymorphonuclear leukocytes (PMNs) at the inflammatory site and an increase in TIM-4 expression on mononuclear phagocytes. Currently, several small-molecule p38 MAPK inhibitors have completed Phase I clinical trials, demonstrating good safety profiles (De Maeyer et al., 2020). Losmapimod is also undergoing Phase III clinical trials for facioscapulohumeral muscular dystrophy (NCT05397470) (Anand et al., 2011; Patnaik et al., 2016).

### 4.2 Biologic agents

Enhanced efferocytosis can reverse hypoxia in murine atherosclerosis, prevent necrotic core expansion (Marsch et al., 2014), and thus treat macrovascular complications of diabetes. Reducing the “do not eat me” signal is a new strategy for atherosclerosis treatment, with some drugs already researched or approved for clearing ACs, such as CD47 antibodies and PD1-/PD-L1 antibodies (Chen et al., 2021). A wealth of data has identified the TAM RTK family as a promising therapeutic target due to its multiple roles in cancer cell survival, metastasis, treatment resistance, and anti-tumor immune suppression. TAM receptor tyrosine kinases play an indispensable role in efferocytosis, the process in which apoptotic cells are engulfed and cleared by macrophages and other phagocytic cells (Scott et al., 2001; Seitz et al., 2007). This prospect has driven the development of various TAM-targeted agents for translational applications, many of which have progressed to clinical testing. Antibody-drug conjugates and decoy receptor fusion proteins have shown significant efficacy in enhancing efferocytosis in tumor cells (DeRyckere et al., 2023), and advancements in enhancing efferocytosis in cancer treatment have provided new directions for research into the treatment of diabetes and its complications.

### 4.3 Nanotechnology

Nanoparticles are nanoscale particulate structures that exhibit distinctive physicochemical and biological characteristics, enabling their application as versatile diagnostic agents and therapeutic modalities (Batista et al., 2015). Some researchers have used various regulatory mediators and agonists to enhance efferocytosis and promote the resolution of inflammation, which has been confirmed in many animal models. Nanoparticles can conveniently package various molecules that alter macrophage function, including siRNA, mRNA, and small molecule inhibitors, thus nanotechnology platforms have advantages in improving macrophage targeting at the lesion site over other technologies (Chen et al., 2022). For example, macrophage-targeted nanoparticles carrying siRNA against Ca2^+^/calmodulin-dependent protein kinase γ (CaMKIIγ), a plaque instability protein activated in late-stage human and mouse plaque macrophages, can mitigate necrotic core expansion by enhancing MerTK-mediated efferocytosis. Similarly, macrophage-targeted single-walled carbon nanotubes loaded with CD47-SIRPα signaling inhibitors can enhance efferocytosis in lesion macrophages and reduce atherosclerosis (Flores et al., 2020). ​Nanoparticles targeting processes associated with macrophage foam cells have been created, such as those that enhance reverse cholesterol transport or suppress LDL oxidation to limit foam cell development (Lewis et al., 2016).​The widespread acceptance of COVID mRNA vaccines has paved the way for exploring mRNA-based therapies. In diabetic mice, the levels of miR-126 are suppressed. A rational intervention for such mice could involve delivering miR-202 to areas burdened with apoptotic debris, such as diabetic wounds, using chitosan modified with nanoparticles or REDV peptides (Zhou et al., 2016). Although nanotechnology has demonstrated promising targeted therapeutic effects and partially mitigates systemic adverse reactions associated with conventional drug administration (Batista et al., 2015), current nanoparticle formulations still lack sufficient selectivity for target cells and intended organs (Smith and Gambhir, 2017; Flores et al., 2019). Furthermore, the clinical safety profile of these nanoscale drug delivery systems requires comprehensive evaluation. These inherent limitations explain why clinically approved nanotherapies remain relatively scarce to date. Nevertheless, this field continues to show substantial potential for advancing precision medicine through continued research and technological refinement.

## 5 Conclusion

In summary, efferocytosis induces the rapid clearance of ACs, preventing secondary necrosis and the release of pro-inflammatory factors, playing a crucial role in maintaining organismal homeostasis. Defects in efferocytosis can lead to homeostatic imbalance, resulting in the onset of diseases. Various types of therapeutic approaches have demonstrated the effectiveness of efferocytosis treatment in cellular and animal models, indicating its potential for treating human pathologies. In recent years, PS receptors on phagocytes have been extensively investigated in the context of cancer therapy, with drugs targeting these receptors showing promising results (Mehrotra and Ravichandran, 2022). The oral or topical administration of endogenous metabolic mediators has been explored as a therapeutic intervention for various other pathologies through mechanisms distinct from endocytosis (Harrison et al., 2021). The deficiency of efferocytosis is closely associated with the pathophysiology of diabetes and its vascular complications, although the molecular signals related to efferocytosis have not been fully elucidated. Further extensive and in-depth research is required to clarify the pathophysiological mechanisms, providing a theoretical basis for efferocytosis-based therapies to reduce potential risks and vascular complications of diabetes.
